# Enhanced cognitive processing by viewing snakes in children with autism spectrum disorder. A preliminary study

**DOI:** 10.1186/s40359-019-0352-6

**Published:** 2019-11-27

**Authors:** Marine Grandgeorge, Alban Lemasson, Martine Hausberger, Hiroki Koda, Nobuo Masataka

**Affiliations:** 1 Centre de Ressources Autisme, CHRU of Brest, Hospital of Bohars, Bohars, France; 2 Marine Grandgeorge, Université de Rennes 1, Ethologie Animale et Humaine, EthoS, UMR 6552, CNRS, Université Caen Normandie, Paimpont, France; 3 CNRS, Univ Rennes, Normandie Univ, EthoS (Éthologie animale et humaine), UMR 6552, F-35380 Paimpont, France; 4 Primate Research Institute, Kyoto University, Kanrin, Inuyama, Aichi Japan

**Keywords:** Snake fear, Emotion, Stroop effect, Autism spectrum disorder, Anxiety

## Abstract

**Background:**

Prioritization of the processing of threatening stimuli induces deleterious effects on task performance. However, emotion evoked by viewing images of snakes exerts a facilitating effect upon making judgments of their color in neurotypical adults and schoolchildren. We attempted to confirm this in school and preschool children with and without Autism Spectrum Disorder (ASD).

**Methods:**

Forty French children participated and corresponded to two age groups: a group of schoolchildren and a group of preschool children, each group including 10 children with typical development and 10 children with ASD. Each participant was exposed to 120 trials composed of 20 photographs of snakes and 20 photographs of flowers, each of which appeared 3 times (in red, green and blue). Participants were asked to indicate the color of each image as quickly as possible via key-press. A three-way analysis of variance test for reaction time (RT) considering image type (IMAGE), participant group (PARTICIPANT), and age (AGE) as main effects and its interaction terms was performed for each subject.

**Results:**

When the reaction time required to respond to presented stimuli was measured, schoolchildren tended to respond faster when stimuli were snake images than when stimuli were flower images whether the children had or did not have ASD. For the 5-to-6-year-old preschool participants, the difference between reaction time for the color-naming of snake images and flower images was ambiguous overall.

**Conclusions:**

There were possible odd color-specific effects in children with ASD when images were presented to the children in green. Implications of the findings are argued with respect to active avoidance or attraction as one of the behavioral characteristics commonly noted in children with ASD.

## Background

To what extent does our performance depend on how we feel? Attempts to answer this question have involved research both on emotion and on cognition. So far, unfortunately, it has generally been assumed conventionally, but with little experimental evidence, that deleterious effects on task performance are a consequence of prioritization of the processing of threatening stimuli [1–3]. More recently, however, some evidence has been presented that challenges this notion. For example, a prior exposure to a fearful face picture enhanced subsequent visual processing [4]. Moreover, in a recent study [5], adults and schoolchildren needed to name the color of images of snakes and flowers using the pictorial emotional Stroop paradigm [6]. Reaction time (RT) to answer was significantly shorter for snake images than for flower images. This was quite surprising, as snake stimuli had a robust attentional bias, in opposition to the reasoning believed in traditional psychobiological studies [7, 8]. In studies such as the one cited above showing a short reaction time to answer the color of snakes, negative emotions such as fear may have an enhancing effect on cognitive processing of stimuli so long as the stimuli are evolutionally relevant threatening ones.

All previous research about such an enhancing effect concerned individuals with typical development. To date, no such attempts have been reported in atypical populations, such as people with autism spectrum disorder (ASD), who need to cope with particular atypical functioning of emotion and cognition e.g. [9]. Although ASD is not classified as an anxiety disorder, it is associated with comorbid anxiety [10, 11]. Indeed, people with ASD were reported to display more anxiety than people with typical development (34–64% versus 5–20% [12], and around 40% displayed atypical and intense fears [13]. For example, children with ASD are more afraid of animals than children with typical development [14]. These fears may interfere significantly with functioning, and thus have a great impact on the daily life as well as on the family network of people with ASD [15], and consequently they often lead to social withdrawal and avoidance [16]. As Mayes et al. [12] explained, “children with ASD may avoid necessary life situations (e.g., refusing to go to school because there may be a fire drill) or be in a constant state of anxiety and unable to function optimally because of their fears”. These situations may lead to strong negative emotional states, e.g., anxiety crises. Moreover, sensorial alterations are present in the core of ASD, for example, these people can be attracted or repulsed by visual stimuli [17] that do not cause such effects in typical development, e.g., preferred or avoided color(s) [18] or color obsession [19, 20]. Likewise, many descriptions of people with ASD showed atypical processing of emotional stimuli [21], though impairments may be more evident when tests involve recognition of complex rather than basic emotions [22]. Similarly, in complex tasks, several studies have found that threatening targets presented during the attentional blink are spared (accurately detected) relative to neutral targets, but it appears that ASD and high levels of autistic traits are linked to an absence of the sparing described above, suggesting that the emotional information is processed differently by people with ASD [23–25]. Indeed, the results of a recent study revealed that attentional bias toward snakes that was observed in a visual search task was enhanced in children with ASD compared to typically developing (TD) children [26], suggesting that the fear response is more extreme in children with ASD. However, to date, the literature gives little information about the cognitive processing involved, even if such data exist for children with typical development. In the case of an emergency, it would be adaptive if the cognitive processing would work effectively in NT children. And if such an emergency were experienced with more anxiety in children with ASD, one could argue that their more extreme fear response would adaptively enhance cognitive processing in them.

The present study was undertaken based on the paradigm employed by these previous studies, and was extended to children with ASD as participants. We hypothesized that negative emotion may have a stronger effect on cognitive processing in ASD children. In our current study, we used snake images and flower images as biologically relevant threatening stimuli and biologically attracting stimuli. If the cognitive processing of the colors of snake images as evolutionally relevant threatening stimuli is enhanced by emotion (perhaps fear) induced by looking at images, such facilitating effects should be similarly robust, and probably higher, in children with ASD than in children without ASD. To test this hypothesis, we included children with and without ASD, from two different age sub-groups. In addition to schoolchildren whose ages were comparable to those of the participants in the previous study (12 years on average) [5], a younger group of preschool children (5 to 6 years) were also included. As the observed facilitation of color-naming of the snake images was less robust in the children than in adults in that previous study [27], we attempted to investigate a developmental aspect of the phenomenon under study by comparing the results of the present experiment between these two age-groups of the children with and without ASD.

## Methods

This study and experimental protocols respected the principles of the Declaration of Helsinki and the Guide for Experimentation with Humans. The international research was submitted and approved by the Institutional Ethics Committee of the Primate Research Institute, Kyoto University (#2011–150). According to French laws at the time of the experiment (*Loi Huriet n°88–1138, 20/12/1988, revision in 2004*), considering that this research was classified as non-interventional research, no additional ethical review board was needed. All parents of all participants involved in the study gave the authors their written informed consent.

## Participants

The current study was undertaken with 40 French children of two age groups, a group of schoolchildren and a group of preschool children, each group including 10 TD children and 10 children with ASD. All were right-handed children. Recruitment was done in schools and institutions in the western part of France, and participants were randomly chosen across volunteers (for a child to be included as a volunteer, their parents had to give written informed consent). None of the children met any diagnostic criterion for having color vision deficiency. The age of the children in the “schoolchildren group” was over 7 years (*M* ± *SD* = 12.9 ± 2.514 for the TD children (6 boys and 4 girls) and 12.1 ± 3.414 for the children with ASD (9 boys and 1 girl), *t* [18] = 0.597, *p =* 0.558). All children included in the “schoolchildren group” group attended school regularly. The age of the children in the “preschool children group” was between 5 and 7 (*M* ± *SD* = 6.3 ± 0.483 for the TD children (6 boys and 4 girls) and 5.8 ± 0.789 for the children with ASD (8 boys and 2 girls), *t* [18] = 1.709, *p =* 0.105).

The diagnosis of autism of each child with ASD was made according to International Classification of Diseases 10th edition (or ICD-10) [28] as well as Diagnostic and Statistical Manual of Mental Disorders 4th edition (or DSM-IV) [29] and done by an independent child psychiatrist based on direct clinical observation. All diagnoses were also completed using the Autism Diagnostic Interview-Revised (ADI-R) [30]. This parental interview was extensive and semi-structured and conducted by an independent psychiatrist. It offers information about “the presence of verbal language skills, defined as daily, functional and comprehensive use of spontaneous phrases of at least three words and occasionally a verb” [30]. In our sample, five participants included in the group of preschool children with ASD were diagnosed as F84.0, four as F84.9, and the other as F84.8 on the basis of such criteria. Three included in the group of schoolchildren with ASD were diagnosed as F84.0, five as F84.5, and the remaining two as F84.9.

Information about intellectual quotient (IQ) score was available for 15 of the 20 children with ASD (mean score: 90.0, range: 70–116). Concerning four of the remaining five children, we were notified by the psychiatrists that their IQ scores were above 70. We were notified that the score was slightly below 70 for the remaining child. All of the participants with ASD were found to express verbal language. None of the participants included in the groups of TD children met any diagnostic criterion for autism or other pervasive developmental disorders, according to school staff.

## Procedure

**Fig. 1:**
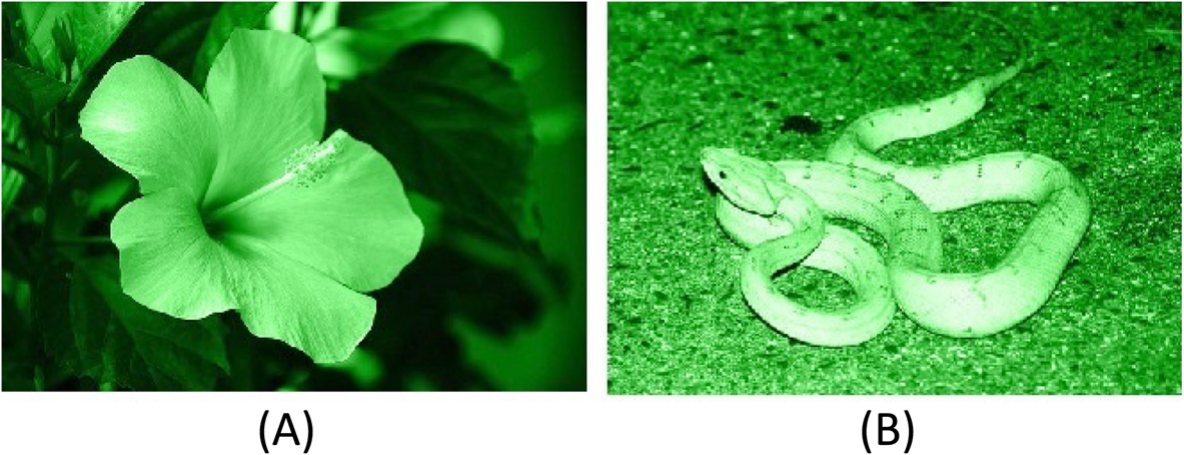
Examples of the stimuli used in the experiment. **a** image of flower in green and **b** image of snake in green. Both photos were taken by one of the authors of the current report

Stimuli consisting of 20 photographs of snakes and 20 photographs of flowers were prepared (example in Fig. 1). Each of the 40 photographs appeared 3 times – once in red, once in green, and once in blue – for a total of 120 experimental trials. These three colors were chosen because they have been extensively used as stimuli in previous psychological research about color perception [31–34]. Color-filtered images were created by performing several steps. First, all color content was removed from the photographs, leading to black and white images. Then, we performed subsequent color balance manipulation along three different dimensions that transformed the midtones, shadows and highlights of each grayscale image, thus preserving luminosity. *Y*, *x*, *y* co-ordinates (means (SDs)) of the three colors (CIE 1931) were 29.5 (3.2), .51 (.07), .34 (.05) for red, 31.4 (4.0), .31 (.05), .45 (.11) for green, and 26.4 (4.7), .18 (.05), .15 (.11) for blue. Each stimulus was presented against a black background.

A 22-in. monitor connected with a personal computer was placed on a table. The experimenter was not aware of the research goal. His presence was limited to instructing the participant about the experiment. In this study, we used a novel adapted single-trial version of the pictorial emotional Stroop task [31]. Participants would see a series of color-filtered photographs (size: 30 cm × 40 cm). They needed to designate the color of each image as quickly as possible, ignoring the content of each photograph. For that, they used a key-press and put each of the index finger (i.e., click if the photograph was red), middle finger (i.e., click if the photograph was green) and the third finger (i.e., click if the photograph was blue) of the right hand upon three different keys on the external numeric keypad. The relationship between the key’s position on the keypad and the color was randomized and counterbalanced across the participants so that the participants might be told to press the middle finger to indicate that the picture was red under one of the other two conditions, and to press the third finger under the remaining condition.

No experiment began without assuring that the participants had understood the instructions given. To assure this, the parents of the children with ASD were asked to confirm that their children had heard and understood the instructions.

Both practice and experimental trials consisted of three events: (1) a white fixation cross, which appeared at the center of the screen for 1 s, (2) the stimulus (picture), and (3) a blank black screen (duration 0.8 s). Each stimulus remained on the screen until the participant pressed one of the three keys, and this duration was recorded as RT in each trial. Fifteen practice trials and 120 experimental trials were randomized in advance. For the practice trials, one random sequence was generated.

For analyses, we performed a three-way analysis of variance test for reaction time data (RT) considering image type (IMAGE), participant group (PARTICIPANT), and age (AGE) as main effects and its interaction terms nested within subject ID, using “*ezANOVA*” methods of the “*ez*” library freely available for R, version 3.5.1. It may also possible to include a random factor for items but these models can be tricky to fit, that why we did not used it here.

## Results

**Fig. 2:**
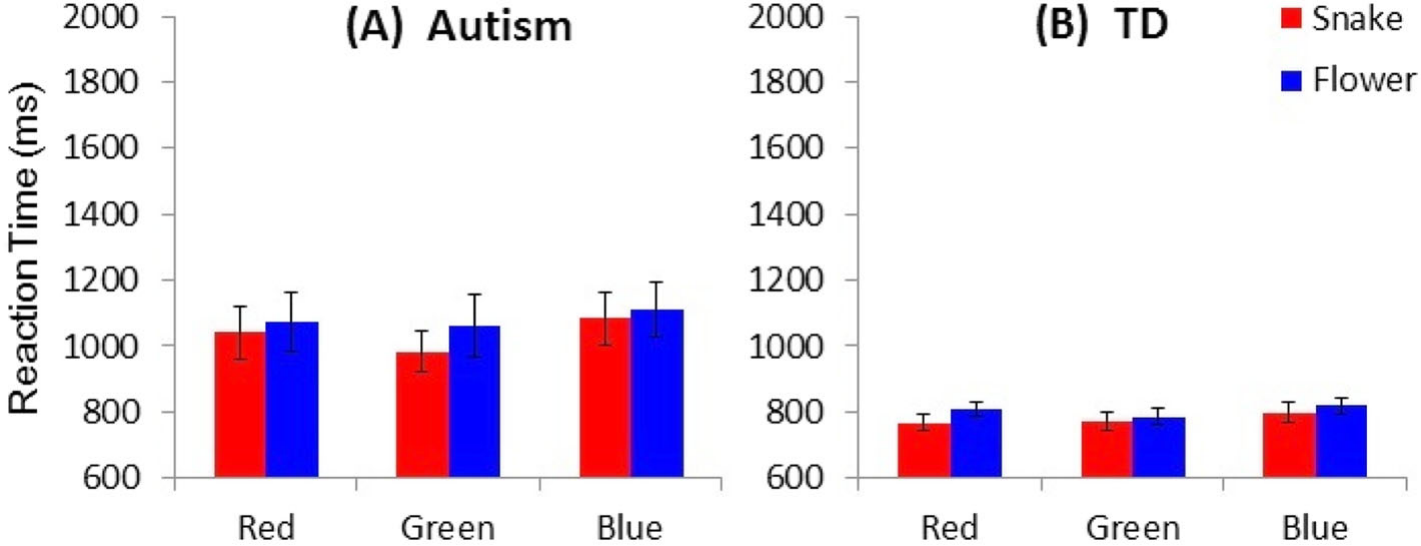
Mean reaction time (RT) of the participant schoolchildren with autism spectrum disorder (**a**) and without the disorder (**b**) across the six variations of stimuli. Error bars represent SDs

**Fig. 3:**
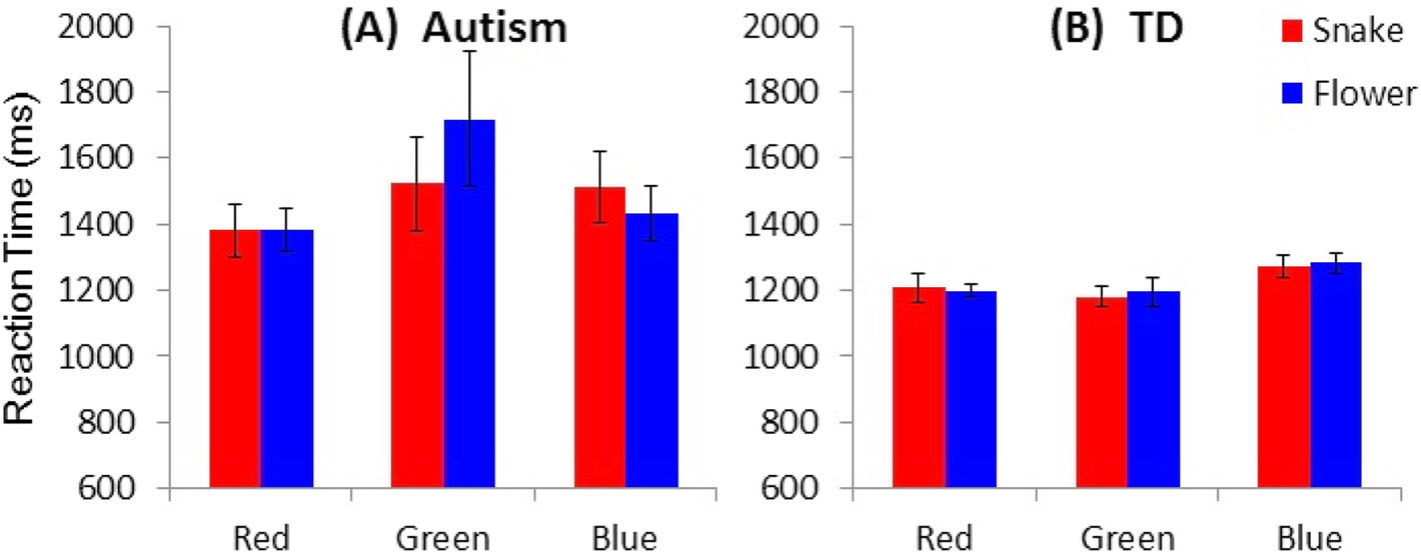
Mean reaction time (RT) of the participant preschool children with autism spectrum disorder (**a**) and without the disorder (**b**) across the six variations of stimuli. Error bars represent SDs

Figure 2 shows mean RTs of the schoolchildren with and without ASD to the six different stimuli, and Fig. 3 shows mean RTs of the 5- to 6-year-old preschool children participants with and without ASD to the six different stimuli. When the collected data were analysed by a 2 (with/without ASD, PARTICIPANT) × 2 (snake/flower, IMAGE) × 2 (preschool/school, AGE) ANOVA (analysis of variance), all of the three main effects were statistically significant (*F* (1, 36) = 16.553, *p* = 0.000246, *η*_*G*_^*2*^ = 0.309 for PARTICIPANT; *F* (1, 36) = 6.9846, *p* = 0.01209, *η*_*G*_^*2*^ = 0.00536 for IMAGE; *F* (1, 36) = 42.88, *p* = 0.000000013, *η*_*G*_^*2*^ = 0.537 for AGE). By contrast, none of the interaction effects were significant. The interaction between PARTICIPANT and IMAGE was not significant (*F* (1, 36) = 1.34, *p* = 0.253, *η*_*G*_^*2*^ = 0.00104). The interaction between PARTICIPANT and AGE was not significant either (*F* (1, 36) = 0.000174, *p* = 0.9895, *η*_*G*_^*2*^ = 0.0000047), and neither was that between IMAGE and AGE (*F* (1, 36) = 0.308, *p* = 0.582, *η*_*G*_^*2*^ = 0.000238). The interaction among PARTICIPANT, IMAGE and AGE was not significant (*F* (1, 36) = 0.0818, *p* = 0.777, *η*_*G*_^*2*^ = 0.0000631).

Overall, children with ASD responded to the presented stimuli more slowly than the TD children regardless of age. In both of these participant groups, RTs to the images of snakes were shorter than RTs to the images of flowers. In addition, schoolchildren consistently responded to the presented stimuli more quickly than preschool children.

Next, we subsequently analyzed the influence of stimulus colours separately for each of age class (school or preschool children). When the collected data for schoolchildren were analyzed by a 2 (with/without ASD, PARTICIPANT) × 2 (snake/flower, IMAGE) × 3 (red/green/blue, COLOR) ANOVA (analysis of variance), all of the three main effects were statistically significant (*F* (1, 18) = 10.046, *p* = 0.005, *η*_*G*_^*2*^ = 0.345 for PARTICIPANT; *F* (1, 18) = 11.813, *p* = 0.003, *η*_*G*_^*2*^ = 0.00925 for IMAGE; *F* (2, 36) = 8.825, *p* = 0.001, *η*_*G*_^*2*^ = 0.013 for COLOR). The interaction between PARTICIPANT and IMAGE was not significant (*F* (1, 18) = 0.884, *p* = 0.360, *η*_*G*_^*2*^ = 0.000698). The interaction between PARTICIPANT and COLOR was not significant, either (*F* (2, 36) = 1.437, *p* = 0.251, *η*_*G*_^*2*^ = 0.00227), and neither was that between IMAGE and COLOR (*F* (2, 36) = 0.772, *p* = 0.470, *η*_*G*_^*2*^ = 0.000649). The interaction among PARTICIPANT, IMAGE and COLOR was not significant (*F* (2, 36) = 1.867, *p* = 0.169, *η*_*G*_^*2*^ = 0.00157).

Likewise, when the collected data for preschool children were analyzed by ANOVA, the main effects were statistically significant for PARTICIPANT (*F* (1, 18) = 7.051, *p* = 0.016, *η*_*G*_^*2*^ = 0.198), but not for IMAGE (*F* (1, 18) = 1.390, *p* = 0.254, *η*_*G*_^*2*^ = 0.00184), or for COLOR (*F* (2, 36) = 1.955, *p* = 0.156, *η*_*G*_^*2*^ = 0.0293). The interaction between PARTICIPANT and IMAGE was not significant (*F* (1, 18) = 0.668, *p* = 0.424, *η*_*G*_^*2*^ = 0.000884). The interaction between PARTICIPANT and COLOR was not significant, either (*F* (2, 36) = 2.920, *p* = 0.067, *η*_*G*_^*2*^ = 0.0431). However, the interaction between IMAGE and COLOR tended to be significant (*F* (2, 36) = 0.196, *p* = 0.053, *η*_*G*_^*2*^ = 0.0120). The interaction among PARTICIPANT, IMAGE and COLOR also tended to be significant (*F* (2, 36) = 2.777, *p* = 0.076, *η*_*G*_^*2*^ = 0.0105).

Overall, the schoolchildren with ASD responded to the presented stimuli more slowly than the TD schoolchildren. In both of these participant groups, RTs to the images of snakes were shorter than RTs to the images of flowers. Regarding the six different stimuli, the TD schoolchildren responded most rapidly to the image of the red snake and most slowly to the image of the blue flower, whereas the schoolchildren with ASD responded most rapidly to the image of the green snake. Among the three stimuli of the flower images, the schoolchildren with ASD also responded most rapidly to the image of the green flower. By contrast, as in the case of schoolchildren with ASD, the preschool children with ASD responded to the presented stimuli more slowly than the TD preschool children. Subsequent analyses of simple main effects (Bonferroni correction), which were performed because of the tendency toward significant interactions between IMAGE and COLOR and among PARTICIPANT, IMAGE and COLOR, revealed that the RT to flower images when they were presented in green was shorter than the RTs to the other five stimulus images in the preschool children with ASD (*p*s < 0.001) whereas RTs did not differ among any of the six stimuli in the TD preschool children (*p*s > 0.10).

## Discussion

The results obtained here in the experiment with TD schoolchildren clearly provided evidence that confirmed the previous findings [5] that viewing images of snakes accelerated making judgments of their color. Regarding the schoolchildren with ASD, too, the current study revealed that the emotion evoked by viewing snake images exerted a facilitating effect upon making judgments of their color. Although overall RTs were longer for the children with ASD than for the TD children, this effect appeared to be as robust in the children with ASD as that in the TD children.

Thus, our results did not seem to support the hypothesis that there is no enhancing effect on cognitive processing in ASD children when compared to children without ASD. Certainly, ASD children are known to be more fearful and phobic than TD children [12]. However, virtually all previous research did not take into consideration whether the threatening stimuli that evoke fear in ASD children are biologically and evolutionally relevant or not. Moreover, in our current study, we used snake images and flower images as biologically relevant threatening stimuli and biologically attractive stimuli, leading to results in ASD children equivalent to those in TD children. One could argue that this finding may involve the question of survival advantage. Thus, the cognitive mechanism of fearful objects may be essentially identical whether children are children with or without ASD. For example, recently, a study reported enhanced attentional bias toward snakes in 8- to 10-year-old children with ASD on the basis of the results of a snake-detection study [26]. Indeed, according to the neurodiversity hypothesis, ASD is not pathological but a part of normal human variation. If so, it would not be surprising that children with ASD are as adaptively predisposed as NT children. Given this fact, the current findings are also in accord with the results of a previous study [35] that showed the acceleration of color-naming in neurotypical students on exposure to an anxiety-producing film, and indicate that children with ASD are no less adept at avoidance than TD children when exposed to evolutionally dangerous stimuli as an adaptive response under such circumstances.

Concerning the data collected from the preschool children, the difference between RTs to the snake images and those to the flower images was ambiguous overall. The task performance of the preschool TD children was not robustly influenced by the color of the presented images. There is accumulating evidence indicating that a stimulus presented in red draws attention more readily than the same stimulus presented in other colors [5, 36–38]. This notion was supported by the results obtained for the TD schoolchildren that the RT required to name the color of an image was shortest when the image of red snakes was presented (red snake effect) and longest when the image of blue flowers was presented, but not by the results obtained for the younger TD children. As an explanation for the age difference reflected in these results, one can reason that there is a difference of the cognitive strategy for snake detection adopted between these two age groups. The results of a previous study [39] using a visual search task revealed that preschool children detected a snake image among flower images faster when the images were in color than when they were in gray-scale, whereas how rapidly adults detected a snake image as the target did not differ whether the presented stimuli were in color or in gray-scale. The authors of that study concluded that young children selectively attended to the color when detecting snakes, whereas adults as well as children older than 10 years of age attended selectively to the shape of the snake. The cortical region responsible for the color processing is different from the region for the processing of the shape, neuroanatomically [40, 41]. In fact, the color processing occurs irrelevant to the shape whose processing occurs depending upon the color [42–44]. Therefore, so long as preschool TD children selectively attend to colors of the presented stimuli, RTs to the snake images would not be different from those to the flower images.

On the other hand, it was found in the present study that there might be some unknown odd color-specific effects on the task performance of preschool children with ASD. Their RT to the green images was likely to be shorter for snake images than for flower images (Fig. 1), whereas no such difference was found with regard to blue images or red images. As an explanation for the delayed response to green flowers, one might assume the possibility that such images were confusing the children with ASD given that green was an atypical color for a flower. Another explanation might be their atypical color processing (e.g., attraction to green color [18–20];. Nevertheless, despite such atypical perception, the fact should be noted that the performance was nearly the same in the group of children with ASD and of TD children, overall, in terms of the difference between responses to snake images and flower images.

Certainly, our study as a preliminary one suffered some limitations. First of all, only a limited number of children with ASD were investigated, with a limited set of stimuli. Although our sample was quite small (*n* = 40), we were able to achieve a good pairing of the groups of children, although we did not match them for either verbal or non-verbal IQ. The results might have been more or less different had a larger participant pool been utilized as well as if the participants had been matched for verbal nor non-verbal IQ (e.g., would children with ASD show better task performance as a consequence of their more intense fears?). The fact that children with and without ASD were not matched for either verbal or non-verbal IQ may suggest that children without ASD have higher verbal IQ or better cognitive processing. Assuming that a fear response enhanced 1 unit of cognitive processing in children without ASD, the fear response may have enhanced 2 units of cognitive processing in children with ASD, and hence the equivalent findings were obtained in the current study. In this sense, fear may have had a stronger effect on cognitive processing in children with ASD, i.e., the original hypothesis of this study may have been supported.

In further studies, we should test the responses to other types of images (other negative ones, e.g., spiders, as well as positive or neutral ones) to more deeply explore the cognitive processing and survival advantage theory. The choice of test length in the present study was driven by the characteristics of children with ASD (i.e., their difficulty of maintaining concentration during a long task). Further investigation into this issue will be required in the near future because, for example, the possible roles of factors such as color preference or developmental age that may have been operative here are still not well known.

## Conclusions

Taken together, the results of the current study indicate that the influence of negative emotion upon the cognitive processing in children with ASD is comparable to that in TD children, raising the question of a possible survival advantage. Continuing to explore such research areas would also assist families and professionals to help people with ASD to cope with their emotional and cognitive particularities, such as fears and phobias.

## Data Availability

The datasets used during the current study are available from the corresponding author on reasonable request.

## Abbreviations

ASD: Autism Spectrum Disorders
DSM-IV: Diagnostic and Statistical Manual of Mental Disorders 4th edition
ICD-10: International Classification of Diseases 10th edition
IQ: Intelligence quotient
RT: Reaction time
TD: Typically developing
